# Associations of upper- and lower-limb muscle health components—muscle mass, absolute strength, and muscle-specific strength—with gait speed in community-dwelling older adults

**DOI:** 10.1007/s41999-026-01565-0

**Published:** 2026-07-24

**Authors:** Yosuke Yamada, Aiko Inoue, Kazuhisa Watanabe, Tomihiko Tajima, Chisato Fujisawa, Hitoshi Komiya, Hiroyuki Umegaki

**Affiliations:** 1 Department of Community Healthcare and Geriatrics, Nagoya University Graduate School of Medicine, Nagoya University, 65 Tsurumai-Cho, Showa-Ku, Nagoya, Aichi 466-8550 Japan; 2 Institute of Innovation for Future Society, Nagoya University Graduate School of Medicine, Nagoya, Japan

**Keywords:** Gait speed, Muscle health, Muscle-specific strength, Muscle quality, Community-dwelling older adults

## Abstract

**Purpose:**

Muscle health encompasses multiple components, including muscle mass, absolute strength, and muscle-specific strength (MSS). MSS, defined as strength relative to corresponding muscle mass, has emerged as a marker of muscle health, but its site-specific relevance to mobility remains unclear. Because these components are often examined separately, direct upper- and lower-limb comparisons are needed to clarify which indicators offer the most clinically relevant information on gait speed. We compared upper- and lower-limb muscle mass, absolute strength, and site-specific MSS in community-dwelling older adults.

**Methods:**

This cross-sectional analysis included 284 community-dwelling adults aged ≥ 65 years. Handgrip strength, knee extension strength, arm and leg muscle mass, and usual gait speed were measured. Upper-limb MSS was calculated as handgrip strength/arm muscle mass, and lower-limb MSS as knee extension strength/leg muscle mass. Age-, sex-, and body mass index-adjusted linear regression models examined associations with gait speed and compared model fit.

**Results:**

Mean age was 77.2 years; 77.1% were women. Arm and leg muscle mass were not associated with gait speed. Knee extension strength showed the strongest individual association (standardized *β* = 0.37, 95% confidence interval 0.23–0.51). Upper-limb MSS (*β* = 0.28, 0.15–0.40) and lower-limb MSS (*β* = 0.32, 0.21–0.44) were associated with gait speed. When entered simultaneously, upper-limb MSS (*β* = 0.18, 0.05–0.31) and lower-limb MSS (*β* = 0.27, 0.16–0.39) remained independently associated, and the combined MSS model showed the best fit.

**Conclusion:**

Site-specific MSS, particularly when assessed in both upper and lower limbs, may provide complementary mobility-related information beyond limb muscle mass alone.

**Supplementary Information:**

The online version contains supplementary material available at https://doi.org/10.1007/s41999-026-01565-0.

## Key summary points

**Aim:**

To compare upper- and lower-limb muscle health components, including muscle mass, absolute strength, and site-specific muscle-specific strength, in parallel and determine which indicators are most informative for gait speed in community-dwelling older adults. Muscle-specific strength is a site-specific indicator that reflects how much force can be generated relative to the corresponding muscle mass.

**Findings:**

Limb muscle mass alone was not associated with gait speed, whereas absolute strength and site-specific muscle-specific strength were associated with gait speed. Knee extension strength showed the strongest individual association, and upper- and lower-limb muscle-specific strength remained independently associated with gait speed when entered simultaneously, with the combined model showing the best fit.

**Message:**

Assessing site-specific muscle-specific strength in both upper and lower limbs may provide complementary information on mobility-related muscle health beyond limb muscle mass alone.

**Supplementary Information:**

The online version contains supplementary material available at https://doi.org/10.1007/s41999-026-01565-0.

## Introduction

Gait speed is one of the most interpretable physical function measures in geriatrics and has a strong prognostic validity [1] [2]. It can be assessed easily and reproducibly, and because it is associated with adverse outcomes such as disability [3], institutionalization [4], and mortality [5], it is widely regarded as a “functional vital sign” that reflects overall health status and physiological reserve in older adults [6]. Because usual gait speed is a simple, reproducible, and continuous measure of mobility, it provides a practical functional outcome for examining whether differences in muscle mass, absolute strength, and MSS are reflected in mobility performance [7] [8]. Normal gait speed can be assessed with a short walk, imposing little participant burden and few environmental constraints while predicting adverse outcomes at least as well as more comprehensive assessments [4]. Moreover, because gait speed is a representative indicator of low physical function in sarcopenia diagnostic algorithms, findings on its association with muscle health-related indicators are readily applicable to clinical decision-making [9] [10].

In contrast, the evaluative framework for age-related muscle impairment has shifted markedly over the past decade from a muscle mass-centered approach to a muscle function-centered one. The European Working Group on Sarcopenia in Older People and the Asian Working Group for Sarcopenia placed muscle strength at the core of diagnosis [9] [10], while the Global Leadership Initiative in Sarcopenia positioned muscle-specific strength (MSS), in addition to muscle mass and muscle strength, as a component of sarcopenia and proposed a conceptual framework in which physical function is regarded not as a defining component but as an outcome [11]. Consistent with this direction, the Asian Working Group for Sarcopenia further expanded the perspective on sarcopenia toward the broader concept of muscle health, emphasizing the linkage to clinically meaningful functional outcomes [12].

Against this background, MSS has recently attracted increasing attention [13]. MSS is a site-specific indicator that reflects how much force can be generated relative to the corresponding muscle mass [14], and it may capture age-related declines in muscle function, not simply as a loss of muscle mass but as a reduction in the force-generating capacity per unit of muscle mass [13] [15]. The dissociation between muscle mass and strength may partly reflect neural and muscular factors, including motor unit recruitment, intramuscular coordination, contractile efficiency, and accumulation of non-contractile tissue [15] [16] [17]. Therefore, preserved muscle mass does not necessarily indicate preserved force-generating capacity. Although there is substantial heterogeneity in the definition and measurement of muscle quality [16], in studies involving older adults, functional indicators that normalize muscle strength to the corresponding muscle mass are among the measures most widely used for this purpose [17]. MSS is also more physiologically interpretable than non-site-specific indices such as handgrip strength (HGS) divided by whole-limb muscle mass [18] [19], and it facilitates better consideration of heterogeneity in the muscle mass–strength relationship according to upper versus lower limbs and by sex [20]. Indeed, a recent longitudinal study showed that upper-limb MSS predicted subsequent decline in physical function better than did HGS alone or the muscle quality index, supporting the clinical usefulness of MSS [13].

However, the clinical and research relevance of site-specific MSS remains insufficiently clarified [17] [21]. Clinically, segmental body composition data may be difficult to interpret when limb muscle mass is considered without corresponding strength. A site-specific strength-to-mass indicator may help contextualize whether the measured muscle mass is accompanied by proportional force-generating capacity. From a research perspective, most earlier studies have focused on either upper-limb MSS or lower-limb MSS alone [13] [22] [23], and few have compared upper- and lower-limb muscle mass, absolute strength, and site-specific MSS in parallel within the same cohort [21] [24]. Although HGS is widely used as a simple marker of muscle strength in older adults, it cannot be assumed to fully represent lower-limb strength or mobility-related muscle health. Therefore, whether HGS can serve as an adequate surrogate marker for lower-limb function or mobility remains a topic of debate [25] [26], and it is still unclear whether upper-limb MSS and lower-limb MSS reflect the same physiological information or provide complementary information [21]. Given that recent studies have also shown that muscle strength is more consistently associated with physical function compared with muscle mass alone [27] [28], examining upper- and lower-limb muscle health components within the same analytical framework using physical function as the outcome has practical and research relevance.

Therefore, in the present study, we performed a cross-sectional analysis of the associations of upper- and lower-limb muscle mass, muscle strength, and site-specific MSS with gait speed in community-dwelling older adults and examined which of these indicators was most strongly associated with gait speed.

## Methods

### Study design

In this study, we analyzed the baseline cross-sectional data from a prospective cohort study investigating the association between knee extension strength (KES) and falls among community-dwelling older adults. Participants were recruited from community-dwelling older adults who voluntarily attended community-based health assessment events conducted in collaboration with local municipal governments. The eligibility criteria were age 65 years or older and no need for assistance with activities of daily living (ADLs). Data were collected from September 2023 to October 2025. Participants were excluded if data on muscle strength, muscle mass, or gait speed were missing.

The study was conducted in accordance with the principles of the Declaration of Helsinki. The study protocol was approved by the ethics committee of Nagoya University. Written informed consent was obtained face-to-face from all participants.

### Measurement of muscle strength

HGS was measured twice on each side using a Smedley-type digital grip dynamometer (GRIP-D; Sanka Co., Ltd., Sanjo, Japan). The highest value (kg) among the four measurements was used for analysis. KES was measured twice on each side, using a handheld dynamometer (mTas F-1; Anima Co., Ltd., Chofu, Japan). Participants sat on the edge of a chair with the hip and knee flexed at approximately 90°. The dynamometer pad was placed against the anterior aspect of the distal lower leg, proximal to the ankle joint. Participants were instructed to extend the knee as strongly as possible against the dynamometer. Two trials were performed on each side, and the highest value (kg) among the four measurements was used for analysis.

### Measurement of cognitive function and depressive symptoms

Clinical psychologists, research assistant nurses, or physicians assessed cognitive function using the Mini-Mental State Examination (MMSE) [29]. The authorized Japanese version of the MMSE, obtained through institutional purchase, was used. This test is scored on a scale of 0 to 30, with lower scores indicating more severe cognitive impairment. Depressive symptoms were assessed using the 15-item Geriatric Depression Scale [30], which is scored on a scale of 0 to 15, with higher scores indicating more severe depressive symptoms.

### Measurement of gait speed

Gait speed was assessed using a walkway-based pressure sensor system (Walk Way; Anima Corporation, Tokyo, Japan). Participants were instructed to walk at their usual pace, and gait speed (cm/s) was obtained from the instrumented measurement section.

### Measurement of muscle mass

Muscle mass was assessed using segmental multi-frequency bioelectrical impedance analysis (InBody 430; InBody Japan Inc., Tokyo, Japan). Upper-limb muscle mass was defined as the sum of the muscle masses of both arms, and lower-limb muscle mass was defined as the sum of the muscle masses of both legs.

### Calculation of muscle-specific strength

Upper-limb MSS was calculated as HGS divided by upper-limb muscle mass, and lower-limb MSS was calculated as KES divided by lower-limb muscle mass.

### Other variables

Age, sex, educational attainment, medical history (hypertension, diabetes, dyslipidemia, and heart disease), and basic ADLs were ascertained in person using interviewer-administered questionnaires. Basic ADLs were assessed using the Barthel Index [31], which ranges from 0 to 100, with higher scores indicating greater independence. Body mass index (BMI) was calculated as weight in kilograms divided by height in meters squared.

### Statistical analysis

The participant characteristics are summarized as the mean (standard deviation), median [interquartile range], or number (%), as appropriate. In the present cross-sectional analysis, the outcome of interest was gait speed (cm/s). The main explanatory variables were HGS, upper-limb muscle mass, upper-limb MSS, KES, lower-limb muscle mass, and lower-limb MSS. Associations between each muscle-related indicator and gait speed were examined using linear regression models, with gait speed as the dependent variable. First, a base model including age, sex, and BMI was constructed, and then each muscle-related indicator was added separately to the model. To examine whether upper-limb MSS and lower-limb MSS were independently associated with gait speed, both variables were entered simultaneously into the same multivariable linear regression model and standardized β coefficients and 95% confidence intervals (CIs) were reported. To compare model performance, we examined adjusted R^2^, root mean square error, Akaike information criterion, and Bayesian information criterion. As supplementary analyses, we explored potential effect modification by sex by testing interaction terms with sex for KES, upper-limb MSS, lower-limb MSS, and the combined MSS model. Sex-specific scatterplots were also constructed for upper-limb MSS and lower-limb MSS.

*P* values < 0.05 were considered statistically significant. All analyses were performed using SPSS ver. 31 (IBM Corp., Armonk, NY) and R ver. 4.5.2 (R Foundation for Statistical Computing, Vienna, Austria).

## Results

A total of 314 participants were enrolled in the study. Of these, 30 were excluded because data on muscle strength, muscle mass, or gait speed were missing, leaving 284 participants for the analysis. Among the 284 participants included in the analysis, the mean age was 77.2 (standard deviation 6.0) years, and 219 (77.1%) were women. The mean BMI was 22.7 (3.1) kg/m^2^ (Table 1). Mean gait speed was 117.2 (25.0) cm/s, mean HGS 24.8 (6.7) kg, mean upper-limb muscle mass 3.5 (0.9) kg, mean upper-limb MSS 7.09 (1.32), mean KES 25.8 (8.2) kg, mean lower-limb muscle mass 11.3 (3.3) kg, and mean lower-limb MSS 2.32 (0.57). The participant characteristics stratified by sex are shown in Supplementary Table S1. Men had a higher BMI and a higher prevalence of diabetes and heart disease, while women had a higher prevalence of dyslipidemia. HGS, KES, and arm and leg muscle mass were higher in men, whereas upper- and lower-limb MSS did not differ significantly by sex.

**Table 1:** Participant characteristics

Variable	Overall	Available *n*
*Demographics*		
Age, years	77.2 (6.0)	284
Female, *n* (%)	219 (77.1%)	284
Education, years	12.3 (2.3)	283
BMI, kg/m^2^	22.7 (3.1)	284
*Clinical characteristics*		
Hypertension, *n* (%)	128 (45.1%)	284
Diabetes, *n* (%)	42 (14.8%)	283
Dyslipidemia, *n* (%)	59 (20.8%)	284
Heart disease, *n* (%)	20 (7.0%)	284
*Cognition and function*		
MMSE score	27.4 (2.3)	283
GDS score	2 [1–4]	270
BADL score	100 [100–100]	284
*Gait speed and muscle measures*		
Gait speed, cm/s	117.2 (25.0)	284
HGS, kg	24.8 (6.7)	284
Arm muscle mass, kg (both arms)	3.5 (0.9)	284
Upper-limb MSS (HGS / arm mass)	7.09 (1.32)	284
KES, kg	25.8 (8.2)	284
Leg muscle mass, kg (both legs)	11.3 (3.3)	284
Lower-limb MSS (KES / leg mass)	2.32 (0.57)	284

Values are mean (standard deviation), median [interquartile range], or number (%) unless otherwise indicated. Available n refers to non-missing observations for each variable. *BMI* body mass index, *MSS* muscle-specific strength, *MMSE* mini-mental state examination, *GDS* geriatric depression scale, *BADL* basic activities of daily living, *HGS*, handgrip strength, *KES* knee extension strength

**Fig. 1:**
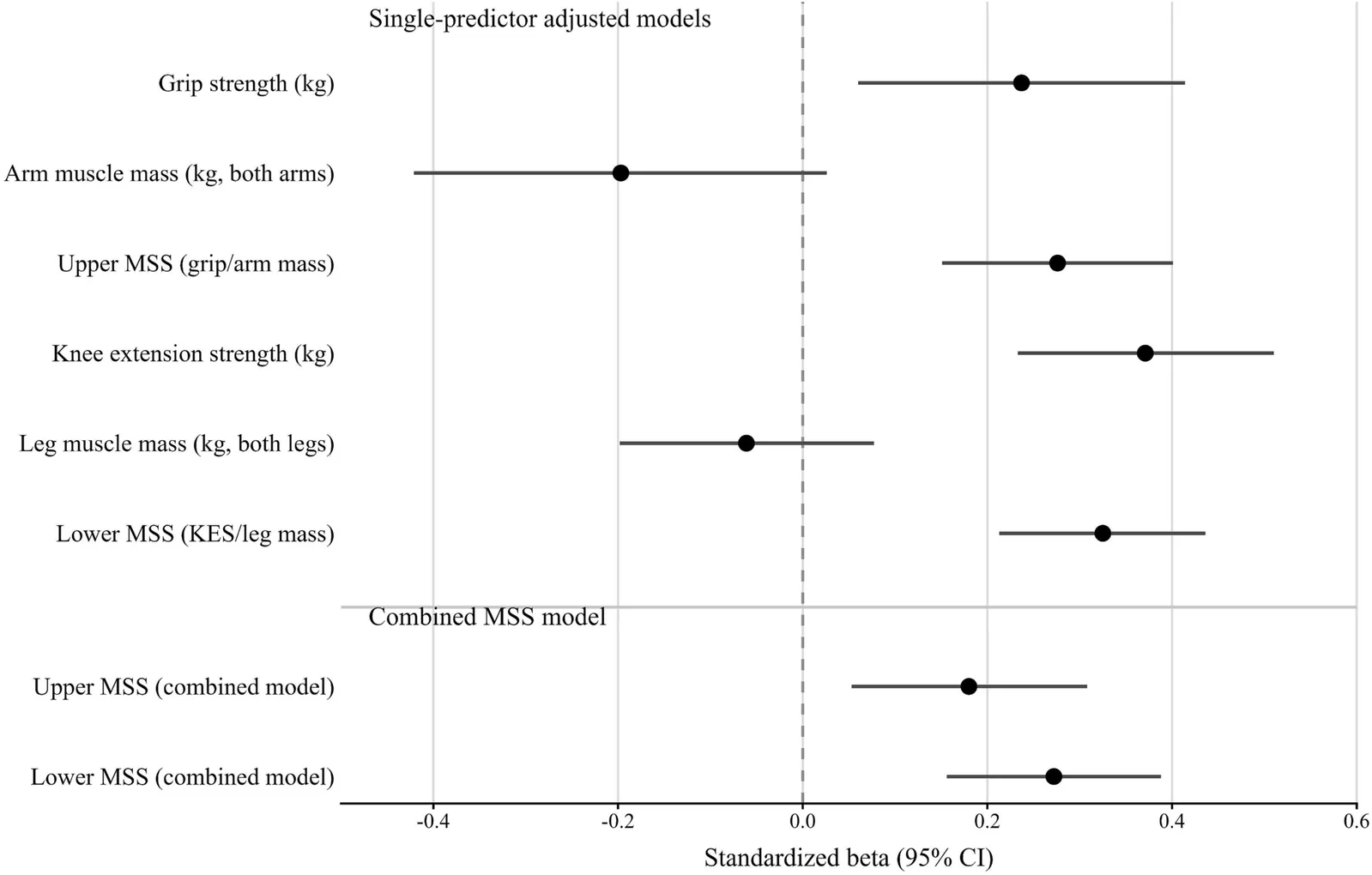
Associations of site-specific muscle measures with gait speed. Forest plot of standardized beta coefficients from single-predictor adjusted models and the mutually adjusted upper- plus lower-MSS model. Comparative model fit is presented in Table 3. HGS, handgrip strength; KES, knee extension strength; MSS, muscle-specific strength

In models adjusted for age, sex, and BMI, both HGS and upper-limb MSS were positively associated with gait speed, whereas upper-limb muscle mass was not (Table 2, Fig. 1). The standardized β coefficients were 0.24 for HGS (95% CI 0.06 to 0.41; *P* = 0.009) and 0.28 for upper-limb MSS (95% CI 0.15 to 0.40; *P* < 0.001). Upper-limb muscle mass was not significantly associated with gait speed (standardized *β* =  − 0.20; 95% CI − 0.42 to 0.03; *P* = 0.083).

**Table 2:** Associations of site-specific muscle measures with gait speed

Predictor	Standardized beta (95% CI)	*P* value	Adjusted R^2^	*N*
*Single-predictor adjusted models*				
HGS (kg)	0.24 (0.06, 0.41)	0.009	0.075	284
Arm muscle mass (kg, both arms)	− 0.20 (− 0.42, 0.03)	0.083	0.062	284
Upper-limb MSS (HGS / arm mass)	0.28 (0.15, 0.40)	< 0.001	0.112	284
KES (kg)	0.37 (0.23, 0.51)	< 0.001	0.138	284
Leg muscle mass (kg, both legs)	− 0.06 (− 0.20, 0.08)	0.385	0.054	284
Lower-limb MSS (KES / leg mass)	0.32 (0.21, 0.44)	< 0.001	0.152	284
*Combined MSS model*				
Upper-limb MSS (combined model)	0.18 (0.05, 0.31)	0.006	0.172	284
Lower-limb MSS (combined model)	0.27 (0.16, 0.39)	< 0.001	0.172	284

Each row shows an age-, sex- and BMI-adjusted linear regression model; the combined MSS model included both upper and lower MSS simultaneously in addition to age, sex, and BMI

*CI* confidence interval, *HGS* handgrip strength, *KES* knee extension strength, *MSS* muscle-specific strength

Indicators related to lower-limb strength showed stronger associations with gait speed. KES was associated with higher gait speed (standardized *β* = 0.37; 95% CI 0.23 to 0.51; *P* < 0.001), and lower-limb MSS was similarly associated with gait speed (standardized *β* = 0.32; 95% CI 0.21 to 0.44; *P* < 0.001). In contrast, lower-limb muscle mass was not associated with gait speed (standardized *β* =  − 0.06; 95% CI − 0.20 to 0.08; *P* = 0.385) (Table 2, Fig. 1). In the combined model, both upper-limb MSS (standardized *β* = 0.18; 95% CI 0.05 to 0.31; *P* = 0.006) and lower-limb MSS (standardized *β* = 0.27; 95% CI 0.16 to 0.39; *P* < 0.001) were independently associated with gait speed.

**Fig. 2:**
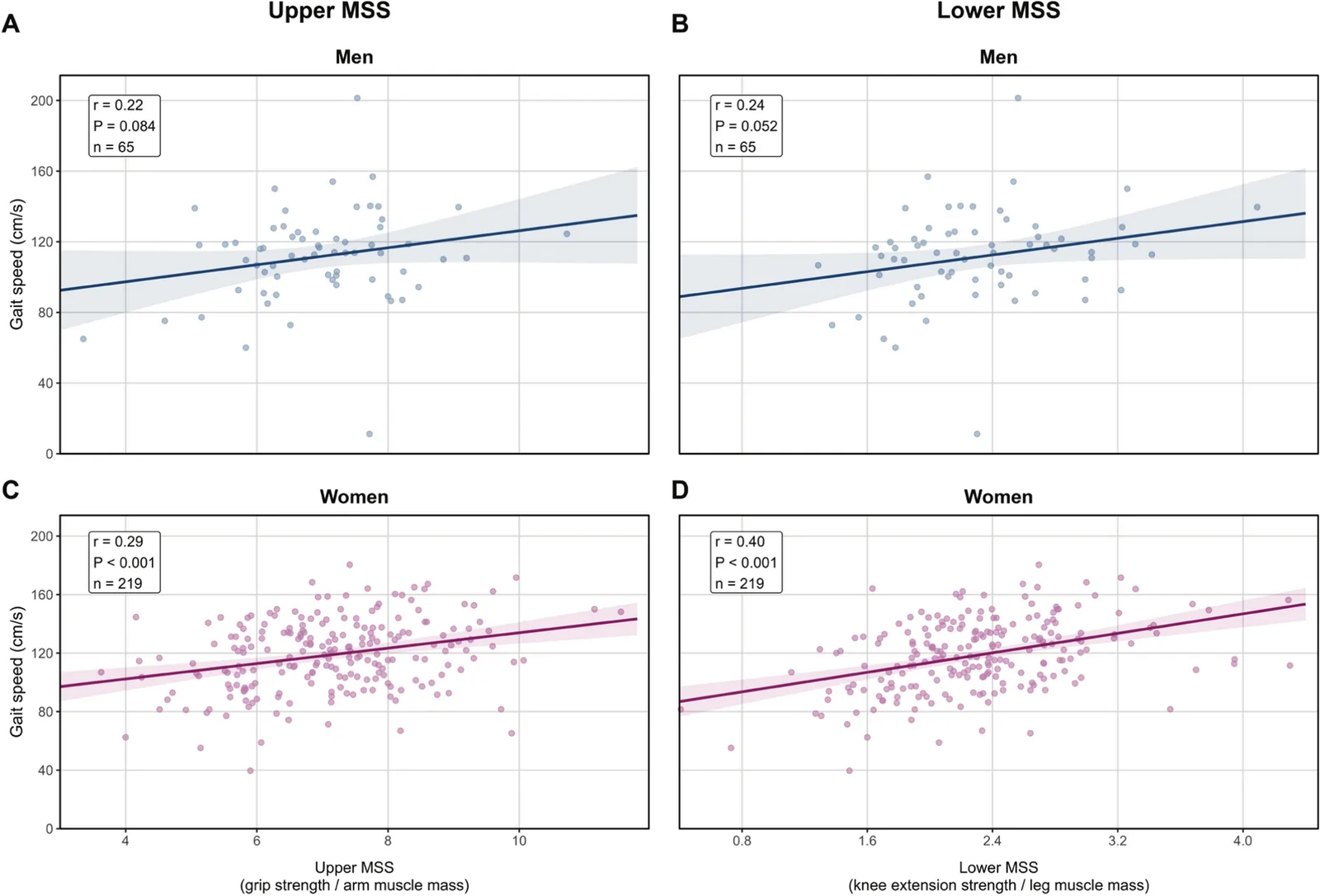
Sex-specific scatter plots of gait speed versus upper- and lower-limb muscle-specific strength. Panels A and B show men; panels C and D show women. Panels A and C show upper-limb MSS (handgrip strength / arm muscle mass), and panels B and D show lower-limb MSS (knee extension strength / leg muscle mass). Solid lines indicate unadjusted least-squares fits with 95% confidence intervals. The scatter plots are intended to show the distribution and approximate linearity of the MSS–gait speed association within each sex; adjusted effect estimates and comparative model fit are presented in Tables 2, 3, respectively. MSS, muscle-specific strength

Model comparisons also showed that models incorporating MSS provided a better fit for gait speed compared with those incorporating limb muscle mass (Table 3). The adjusted R^2^ of the base model including only age, sex, and BMI was 0.055. Adding upper- or lower-limb muscle mass yielded little improvement (adjusted R^2^ 0.062 and 0.054, respectively), whereas the adjusted R^2^ increased to 0.138 with KES, 0.112 with upper-limb MSS, and 0.152 with lower-limb MSS. The combined model including both upper- and lower-limb MSS showed the best performance (adjusted R^2^ 0.172; RMSE 22.51 cm/s; Akaike information criterion 2586.7; Bayesian information criterion 2608.6). In supplementary sensitivity analyses, a sex interaction was observed only for the association between KES and gait speed, with a stronger association in women, whereas no clear interaction was observed for upper-limb MSS, lower-limb MSS, or the combined MSS model (Supplementary Table S2). The sex-specific scatterplots in Fig. 2 show roughly linear positive associations for both MSS measures in both sexes.

**Table 3:** Model comparison for gait speed

Model	N	R^2^	Adjusted R^2^	ΔAdjusted R^2^ vs base	RMSE (cm/s)	AIC	BIC
Base (age, sex, BMI)	284	0.065	0.055	0.000	24.13	2622.2	2636.8
Base + HGS	284	0.088	0.075	0.020	23.84	2617.2	2635.4
Base + arm muscle mass	284	0.075	0.062	0.007	24.00	2621.1	2639.4
Base + upper-limb MSS	284	0.124	0.112	0.057	23.35	2605.6	2623.8
Base + HGS + arm muscle mass	284	0.124	0.108	0.053	23.36	2607.7	2629.6
Base + KES	284	0.150	0.138	0.083	23.01	2597.1	2615.3
Base + leg muscle mass	284	0.067	0.054	-0.001	24.10	2623.4	2641.7
Base + lower-limb MSS	284	0.164	0.152	0.097	22.82	2592.5	2610.8
Base + KES + leg mass	284	0.162	0.146	0.092	22.85	2595.2	2617.1
Base + upper-limb MSS + lower-limb MSS	284	0.186	0.172	0.117	22.51	2586.7	2608.6

The base model included age, sex and BMI. ΔAdjusted R^2^ indicates the increment in adjusted R^2^ relative to the base model

*RMSE* root mean square error, *AIC* Akaike information criterion, *BIC* Bayesian information criterion, *BMI* body mass index, *MSS* muscle-specific strength, *HGS* handgrip strength, *KES* knee extension strength

## Discussion

The study examined the associations of upper- and lower-limb muscle health components, including muscle mass, absolute strength, and site-specific MSS, with gait speed to assess whether upper- and lower-limb MSS provide complementary information. The three main findings of this study are as follows. First, among community-dwelling older adults, limb muscle mass alone was not associated with gait speed, whereas absolute muscle strength and MSS were. Second, among the individual indicators, KES showed the strongest association with gait speed. Third, upper-limb MSS and lower-limb MSS remained independently associated with gait speed even when included simultaneously in the same model, and the model incorporating both showed the best fit. KES showed the strongest individual association with gait speed, which is consistent with the central role of lower-limb strength in walking. MSS adds a strength-to-mass perspective by relating absolute strength to the corresponding limb muscle mass. When upper- and lower-limb MSS were included in the same model, both remained associated with gait speed. This finding suggests that upper- and lower-limb MSS reflect partly distinct aspects of mobility-related muscle health. The best-fitting model still explained only part of the variance in gait speed, supporting the multifactorial nature of mobility in older adults.

These findings are consistent with the ongoing shift from a muscle mass-centered view of sarcopenia to a broader muscle health framework that places greater emphasis on muscle strength and physical function [9] [12]. In earlier studies of older adults, indicators that normalize muscle strength to the corresponding muscle mass [17] have been used as functional measures to capture the discrepancy between the amount of existing muscle tissue and the actual force-generating capacity [16]. The Baltimore Longitudinal Study of Aging found that several muscle quality indices were closely associated with lower-extremity performance [32]. Furthermore, a study of older Japanese outpatients reported that lower-limb muscle quality was independently associated with slow gait speed [22]. In addition, a recent longitudinal study showed that upper-limb MSS predicted a subsequent decline in physical function better than HGS alone or the muscle quality index [13]. The present study extends this line of evidence to gait speed in a community-dwelling cohort and is novel in that it directly compares upper- and lower-limb muscle mass, absolute muscle strength, and MSS within the same cohort.

A particularly important finding of this study is that upper-limb MSS and lower-limb MSS remained independently associated with gait speed even when entered simultaneously in the same model. HGS and quadriceps (knee extension) strength are not necessarily strongly correlated in older adults. Chan et al. reported not only a weak association between the two measures but also that each was independently associated with several health outcomes, including gait speed [33]. Fragala et al. likewise showed that KES was somewhat superior to HGS in predicting slow gait speed, although both were useful for screening for gait decline [34]. Furthermore, Harris-Love et al. demonstrated that, although lower-limb strength is more strongly related to physical function, upper-limb strength is not simply a surrogate for lower-limb strength [35]. The significance of the present study lies in extending this notion—that upper- and lower-limb indicators are not fully interchangeable—from absolute strength measures to site-specific MSS.

One possible explanation for this complementarity is that lower-limb MSS may more strongly reflect a mobility-specific capacity directly involved in weight acceptance, support, and propulsion during gait, whereas upper-limb MSS may reflect a broader neuromuscular status that cannot be fully captured by lower-limb measures alone. Importantly, this does not imply that the upper limbs directly drive gait; rather, it suggests that MSS measured at different anatomical sites can be used to capture distinct aspects of muscle health. Muscle mass does not adequately reflect many of the age-related factors that determine mobility, including neuromuscular activation, motor unit remodeling, intramuscular fat infiltration, fibrosis, tendon properties, and contractile efficiency [36] [37]. Accordingly, the actual force-generating capacity may decline even when limb muscle mass is preserved, and MSS may capture this discrepancy better compared with muscle mass alone [15]. This interpretation is also consistent with findings from a systematic review showing that physical function in older adults is more consistently associated with muscle strength than with muscle mass [27].

These findings have two main implications for clinical assessment and research. Clinically, when segmental body composition data are available, interpreting MSS derived from the corresponding limb muscle mass and absolute muscle strength may provide more informative context than reporting limb muscle mass alone. In particular, lower-limb MSS may serve as a practical adjunctive marker of mobility-related muscle health when both KES and leg muscle mass are already available. From a research perspective, the independent association of upper-limb MSS indicates that upper-limb measures should be regarded as complementary to, rather than interchangeable with, lower-limb measures.

In the supplementary analyses, a sex interaction was suggested only for the association between KES and gait speed, with a stronger association observed in women. In contrast, no clear interaction was observed for lower-limb MSS. This may suggest that raw KES retains more of the sex-related differences in body size and body composition, whereas some of these differences may be attenuated in indices normalized to muscle mass. However, given the limited number of men in the present study, this finding should be interpreted as hypothesis-generating.

This study has several strengths. We compared upper and lower limbs within the same cohort and evaluated muscle mass, absolute muscle strength, and MSS in parallel. In addition, we intentionally kept the analytical strategy simple, which may enhance the interpretability of the findings and their potential clinical applicability. However, several limitations should be acknowledged. First, because this was a cross-sectional analysis, causal relationships cannot be inferred. Second, this was a single community-dwelling cohort with a predominance of women, which limits generalizability. Third, muscle mass was estimated using segmental multi-frequency bioelectrical impedance analysis. BIA-derived segmental muscle mass may be influenced by hydration status and extracellular fluid and cannot distinguish contractile muscle tissue from edema or other non-contractile components. Accordingly, MSS in this study should be interpreted as an operational, BIA-derived, site-specific strength-to-mass indicator rather than as a direct morphological measure of muscle quality. Fourth, residual confounding from unmeasured factors such as physical activity, nutrition, pain, osteoarthritis, and neurological disease cannot be fully excluded.

## Conclusion and implications

In community-dwelling older adults, site-specific MSS, particularly when assessed in both the upper and lower limbs, may provide complementary information on gait speed beyond limb muscle mass alone. The independent associations of upper-limb and lower-limb MSS suggest that these measures capture complementary aspects of muscle health.

## Supplementary Information

Below is the link to the electronic supplementary material.

Supplementary file1 (DOCX 30 KB)

Supplementary file2 (DOCX 30 KB)

## Data Availability

The datasets generated and/or analyzed during the current study are available from the corresponding author upon reasonable request, subject to ethical and privacy restrictions.
