# Supplementary material for: Associations of upper- and lower-limb muscle health components—muscle mass, absolute strength, and muscle-specific strength—with gait speed in community-dwelling older adults
Source: Eur Geriatr Med. 2026 Jul 24;17(5):2535–43. doi: 10.1007/s41999-026-01565-0 (PMC13755018; doi:10.1007/s41999-026-01565-0)
Supplement: Supplementary file 1 [file 41999_2026_1565_MOESM1_ESM.docx]

**Supplementary Table S1. Participant characteristics by sex**

| **Variable** | **Men (n=65)** | **Women (n=219)** | **Available n (Men/Women)** | **P value** |
| --- | --- | --- | --- | --- |
| **Demographics** | | | | |
| Age, years | 76.9 (6.7) | 77.2 (5.8) | 65/219 | 0.728 |
| Education, years | 12.7 (2.9) | 12.2 (2.1) | 65/218 | 0.150 |
| BMI, kg/m^2^ | 23.6 (3.0) | 22.4 (3.1) | 65/219 | 0.006 |
| **Clinical characteristics** | | | | |
| Hypertension, n (%) | 30 (46.2%) | 98 (44.7%) | 65/219 | 0.954 |
| Diabetes, n (%) | 17 (26.2%) | 25 (11.5%) | 65/218 | 0.006 |
| Dyslipidemia, n (%) | 6 (9.2%) | 53 (24.2%) | 65/219 | 0.015 |
| Heart disease, n (%) | 12 (18.5%) | 8 (3.7%) | 65/219 | <0.001 |
| **Cognition, mood and function** | | | | |
| MMSE score | 27.1 (2.4) | 27.5 (2.2) | 65/218 | 0.282 |
| GDS score | 2 [0–4] | 2 [1–5] | 60/210 | 0.062 |
| BADL score | 100 [100–100] | 100 [100–100] | 65/219 | 0.392 |
| **Gait speed and muscle measures** | | | | |
| Gait speed, cm/s | 111.6 (26.4) | 118.8 (24.4) | 65/219 | 0.051 |
| HGS, kg | 33.5 (6.5) | 22.2 (4.0) | 65/219 | <0.001 |
| Arm muscle mass, kg (both arms) | 4.9 (0.8) | 3.2 (0.6) | 65/219 | <0.001 |
| Upper-limb MSS (HGS / arm mass) | 7.0 (1.2) | 7.1 (1.4) | 65/219 | 0.308 |
| KES, kg | 33.5 (9.5) | 23.6 (6.3) | 65/219 | <0.001 |
| Leg muscle mass, kg (both legs) | 14.4 (2.1) | 10.4 (3.0) | 65/219 | <0.001 |
| Lower-limb MSS (KES / leg mass) | 2.3 (0.5) | 2.3 (0.6) | 65/219 | 0.980 |

Values are mean (standard deviation), median [interquartile range], or number (%) unless otherwise indicated. P values were calculated using Welch’s t-test for continuous variables, except for GDS and BADL, which were compared with the Mann–Whitney *U* test. Categorical variables were compared using the chi-square test or Fisher’s exact test as appropriate. Available n refers to non-missing observations in men and women, respectively. BMI, body mass index; MSS, muscle-specific strength; MMSE, Mini-Mental State Examination; GDS, Geriatric Depression Scale; BADL, basic activities of daily living; HGS, handgrip strength; KES, knee extension strength
