# Supplementary material for: Associations of upper- and lower-limb muscle health components—muscle mass, absolute strength, and muscle-specific strength—with gait speed in community-dwelling older adults
Source: Eur Geriatr Med. 2026 Jul 24;17(5):2535–43. doi: 10.1007/s41999-026-01565-0 (PMC13755018; doi:10.1007/s41999-026-01565-0)
Supplement: Supplementary file 2 [file 41999_2026_1565_MOESM2_ESM.docx]

**Supplementary Table S2. Sex interaction sensitivity analyses for associations of muscle measures with gait speed**

Standardised beta coefficients and 95% confidence intervals are shown for sex-stratified models.

| **Predictor** | **Men (n=65) Standardised beta (95% CI)** | **Women (n=219) Standardised beta (95% CI)** | **P for interaction** |
| --- | --- | --- | --- |
| **Single-predictor adjusted models** | | | |
| HGS (kg) | 0.16 (−0.12, 0.44) | 0.19 (0.05, 0.33) | 0.069 |
| Arm muscle mass (kg, both arms) | −0.12 (−0.45, 0.21) | −0.11 (−0.27, 0.06) | 0.688 |
| Upper-limb MSS (HGS / arm mass) | 0.25 (−0.02, 0.51) | 0.27 (0.13, 0.41) | 0.925 |
| KES (kg) | 0.24 (−0.03, 0.52) | 0.38 (0.25, 0.51) | 0.004 |
| Leg muscle mass (kg, both legs) | −0.03 (−0.30, 0.25) | −0.05 (−0.19, 0.08) | 0.639 |
| Lower-limb MSS (KES / leg mass) | 0.28 (0.02, 0.54) | 0.34 (0.22, 0.47) | 0.335 |
| **Mutually adjusted MSS model** | | | |
| Upper-limb MSS (HGS / arm mass) | 0.17 (−0.10, 0.45) | 0.16 (0.02, 0.31) | 0.857 |
| Lower-limb MSS (KES / leg mass) | 0.23 (−0.05, 0.50) | 0.30 (0.17, 0.43) | 0.355 |

Sex-stratified models were adjusted for age and BMI. P for interaction was obtained from pooled models adjusted for age, sex, BMI, the corresponding muscle measure, and a sex-by-predictor interaction term. For the mutually adjusted MSS model, upper MSS and lower MSS were entered simultaneously in both the sex-stratified and pooled interaction models. Standardisation was performed within each sex-specific analytic sample for the sex-stratified models. CI, confidence interval; HGS, handgrip strength; KES, knee extension strength; MSS, muscle-specific strength.
